# 3D modelling of drug-coated balloons for the treatment of calcified superficial femoral arteries

**DOI:** 10.1371/journal.pone.0256783

**Published:** 2021-10-11

**Authors:** Monika Colombo, Anna Corti, Scott Berceli, Francesco Migliavacca, Sean McGinty, Claudio Chiastra

**Affiliations:** 1 Laboratory of Biological Structure Mechanics (LaBS), Department of Chemistry, Materials and Chemical Engineering “Giulio Natta”, Politecnico di Milano, Milan, Italy; 2 Malcom Randall VAMC, Gainesville, Florida, United States of America; 3 Department of Surgery, University of Florida, Gainesville, Florida, United States of America; 4 Department of Biomedical Engineering, University of Glasgow, Glasgow, United Kingdom; 5 PoliTo^BIO^Med Lab, Department of Mechanical and Aerospace Engineering, Politecnico di Torino, Turin, Italy; Medical University Innsbruck, AUSTRIA

## Abstract

**Background/Objectives:**

Drug-coated balloon therapy for diseased superficial femoral arteries remains controversial. Despite its clinical relevance, only a few computational studies based on simplistic two-dimensional models have been proposed to investigate this endovascular therapy to date. This work addresses the aforementioned limitation by analyzing the drug transport and kinetics occurring during drug-coated balloon deployment in a three-dimensional geometry.

**Methods:**

An idealized three-dimensional model of a superficial femoral artery presenting with a calcific plaque and treated with a drug-coated balloon was created to perform transient mass transport simulations. To account for the transport of drug (i.e. paclitaxel) released by the device, a diffusion-reaction equation was implemented by describing the drug bound to specific intracellular receptors through a non-linear, reversible reaction. The following features concerning procedural aspects, pathologies and modelling assumptions were investigated: (i) balloon application time (60–180 seconds); (ii) vessel wall composition (healthy vs. calcified wall); (iii) sequential balloon application; and (iv) drug wash-out by the blood stream vs. coating retention, modeled as exponential decay.

**Results:**

The balloon inflation time impacted both the free and specifically-bound drug concentrations in the vessel wall. The vessel wall composition highly affected the drug concentrations. In particular, the specifically-bound drug concentration was four orders of magnitude lower in the calcific compared with healthy vessel wall portions, primarily as a result of reduced drug diffusion. The sequential application of two drug-coated balloons led to modest differences (~15%) in drug concentration immediately after inflation, which became negligible within 10 minutes. The retention of the balloon coating increased the drug concentration in the vessel wall fourfold.

**Conclusions:**

The overall findings suggest that paclitaxel kinetics may be affected not only by the geometrical and compositional features of the vessel treated with the drug-coated balloon, but also by balloon design characteristics and procedural aspects that should be carefully considered.

## 1. Introduction

The number of individuals suffering from lower limb peripheral artery disease is increasing globally. The incidence of the disease in people aged 25 years and over was reported to be approximately 230 million in 2015, representing a notable increase compared with the estimated 202 million people affected by the disease in 2010 [1]. The pathology is characterized by the presence of atherosclerotic lesions that, by protruding into the arterial lumen, obstruct the blood flow and impair the perfusion of the lower limb [2]. The superficial femoral artery (SFA), the longest arterial vessel of the human body [3], is the location of around half of the peripheral atherosclerotic lesions, due to its morphology and biomechanics [4, 5]. Given the lower risk of complications and the lower level of invasiveness, a primary endovascular revascularization is usually preferred to surgical bypass for most SFA lesions [6]. Despite the ability of self-expandable stents to bear the dynamic load typical of the femoral arteries, the role of these bare-metal devices has become a matter of great controversy because of the high rate of failure due to restenosis (~30%) and stent fracture [7]. Thus, in recent years, drug-coated balloons (DCBs) have emerged as an alternative to the conventional non-medicated angioplasty/stenting treatment of atherosclerotic SFAs.

DCBs are capable of limiting excessive neointima proliferation, by combining the mechanical action of the angioplasty inflation with the therapeutic action of the antiproliferative drug released and transferred into the vessel wall during the intervention [8]. In particular, paclitaxel-eluting balloons have shown promising results, inducing a desirable biological effect, namely the inhibition of cellular proliferation, without the use of permanent implants [9]. Whether by single or sequential balloon inflation, DCBs may enhance the outcome in the primary treatment of SFA restenosis, with lower risk as compared to surgical bypass [10]. Despite the initial promising findings in the case of treatment of lower limb arteries, the safety of paclitaxel-based systems for the SFAs has been questioned, given the high rate of death at 2–5 years following DCB [11]. Moreover, recurrent restenosis remains a persistent problem following DCB use as compared to surgical bypass [12]. For these reasons, further investigations of the DCB mode of action for the treatment of peripheral artery disease are warranted.

Computational modelling has long been recognized as an effective tool to investigate the phenomena of drug transport and kinetics associated with drug-eluting endovascular devices [13, 14]. Through the inclusion of a reaction term within the governing drug transport equations, computational models provide insights into paclitaxel uptake and release with a level of detail difficult or impossible to replicate with *in-vitro* or *in-vivo* methods. Despite the usefulness of computational methodology to assess the safety and the performance of DCBs, to date only a few studies have considered drug transport following release from DCBs [15–18] and only one of these has focused on its application in SFAs. Furthermore, all of these studies are inherently limited by their consideration of a highly-simplified two-dimensional (2D) vessel geometry.

The present work addresses the aforementioned limitation by computationally investigating the efficacy and mode of action of DCBs in a three-dimensional (3D) SFA geometry. In detail, an idealized asymmetric 3D computational model of a diseased SFA treated with a DCB is presented to investigate the impact on the paclitaxel transport and kinetics of the following features: (i) DCB inflation time, which may vary according to the device under consideration and the preferences of the vascular surgeon; (ii) vessel wall material properties; (iii) sequential application of multiple DCBs and; (iv) drug wash-out by the blood stream as opposed to the permanence of DCB coating adhered to the lumen wall.

## 2. Material and methods

### 2.1 Computational model of a diseased SFA treated with a DCB

**Fig 1:**
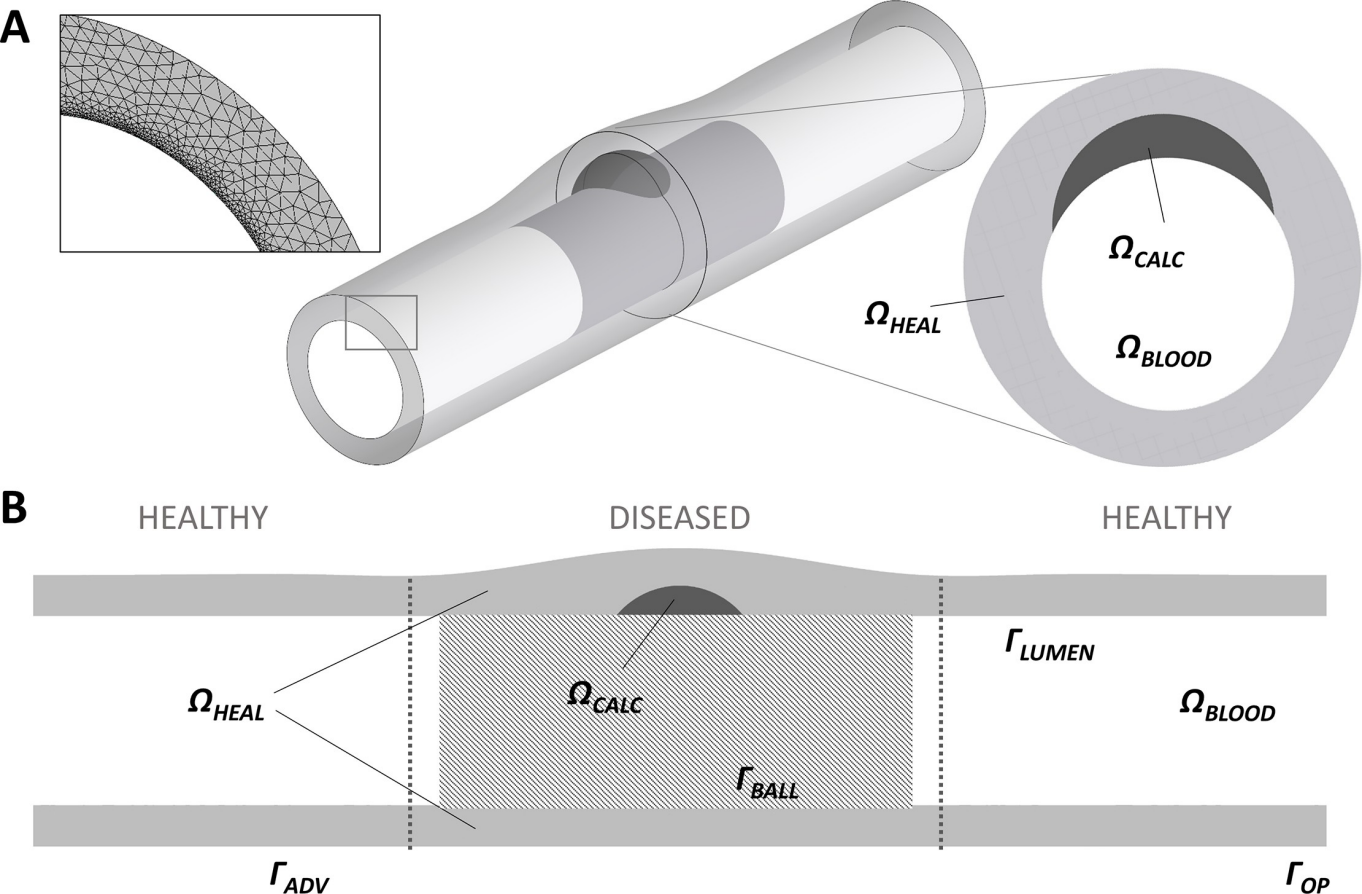
**A)** Geometrical model of a 3D idealized portion of diseased superficial femoral artery (SFA), highlighting the computational grid employed within the wall at the inlet cross-section (on the left) and cross-section of the diseased portion characterized by the presence of a calcific plaque (on the right). **B)** Section along the longitudinal direction of the SFA model, with the definition of the computational domains and boundaries. *Ω*_*HEAL*_: healthy domain; *Ω*_*CALC*_: calcific plaque domain; *Ω*_*BLOOD*_: lumen domain (not meshed); *Γ*_*LUMEN*_: lumen wall; *Γ*_*BALL*_: lumen wall in contact with the balloon; *Γ*_*OP*_: lateral openings of the vessel wall; *Γ*_*ADV*_: adventitial wall.

An idealized 3D model of a diseased SFA treated with a paclitaxel coated balloon was created using the computer aided-design modeler Rhinoceros 3D (v.6, Robert McNeel & Associates, Seattle, WA, USA) (Fig 1), assuming a complete patency (100%) achieved during the balloon inflation, resulting in a constant lumen diameter of 7.3 mm [4]. The vessel model was subdivided in a central diseased portion, which was treated with the DCB, and two healthy portions proximal and distal to the lesion. The diseased portion included a calcific plaque (*Ω*_*CALC*_), causing a lumen area reduction of 40% prior to DCB inflation. In accordance with Glagov’s phenomenon of vascular remodeling [19], the vessel wall presented an increased thickness in the proximity of the atherosclerotic plaque. By considering a thickness of 1.6 mm for the healthy portions (22% of the lumen diameter, as measured in human SFAs through the Doppler ultrasound technique [20]), the thickness of the diseased portion was set equal to 1.9 mm, slightly larger (~18%) than that of the healthy portions. The properties of the calcific plaque *Ω*_*CALC*_ in terms of drug diffusivity (1.99·10^−7^ mm^2^/s) and maximum density of drug binding sites (1.3 mmol/L) were derived from existing literature [21]. The properties of the healthy tissue *Ω*_*HEAL*_, obtained from studies on healthy arterial vessels [18, 22], were assumed to be 5.41·10^−5^ mm^2^/s for the drug diffusivity and 4.2 mmol/L for the maximum density of binding sites.

To simplify the complex interaction between the inflated balloon and the vessel wall, the action of the device was imposed as a boundary condition. According to the instruction for use of the IN.PACT Admiral device (Medtronic, Dublin, Ireland), whose characteristics were representatively employed in this work, a balloon of 7.0x40 mm was considered to match the dimensions of the idealized SFA model.

To perform the mass transport simulations, the geometrical model of the vessel wall was discretized through ICEM CFD (v.18.2, Ansys Inc., Canonsburg, PA, USA) into tetrahedral elements (global minimum element size set to 0.4 mm and local minimum size set to 0.025 mm) and 20 layers of prismatic elements with a gradual smaller size close to the lumen wall *Γ*_*BALL*_ and *Γ*_*LUMEN*_ (with an exponential growth rate of 1.1 and a total height of 0.05 mm). A mesh independence analysis was conducted to choose the mesh size in terms of element size and number of prismatic layers. This analysis was based on the validation of the computational model against the analytical solution of the transient linear diffusion equation in an idealized hollow cylinder (detailed in the S1 File, Section “Analytical validation and sensitivity analysis”), resulting in a final mesh cardinality of ~23 million elements. The blood domain was not included in the analysis and, hence, it was not discretized. Our justification for this is based on the fact that during balloon inflation the blood stream is impeded by the presence of the device itself. Once the device is deflated and removed, blood wash-out may be modeled through the prescription of a boundary condition on the lumen side of the wall, without increasing the computational effort, as described in detail in section 2.2.2.

### 2.2 Computational analysis

#### 2.2.1 Transport equations

**Fig 2:**
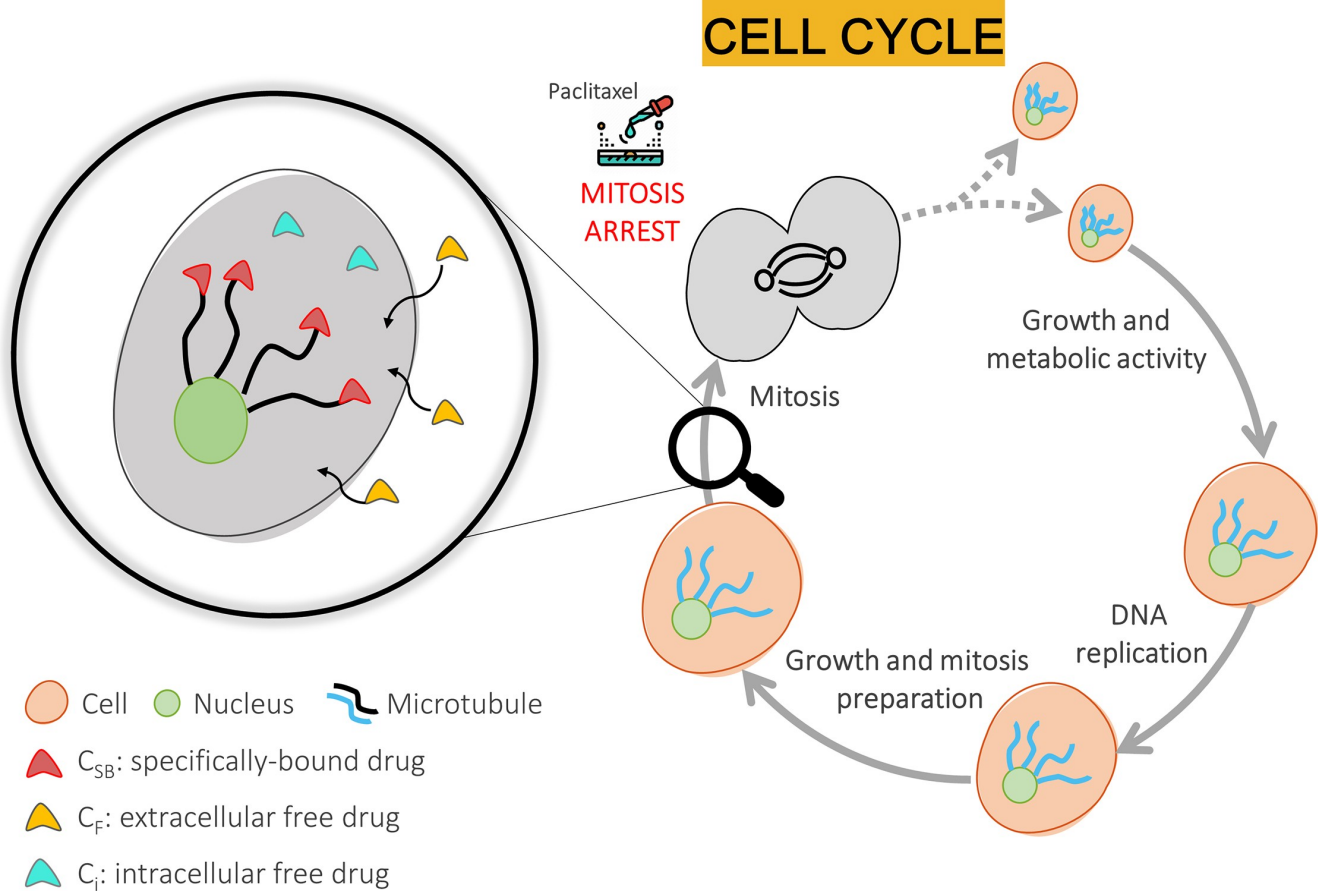
Schematic representation of the biological process occurring during the cell cycle and the current understanding of the action of paclitaxel. After the phase of growth and initial metabolic activity, the cell undergoes the phase of DNA replication and ulterior growth. Once maturity has been reached, the cell prepares for the mitotic process, which in physiological conditions would lead to cell division into two identical daughter cells. However, when the system is subjected to the anti-proliferative paclitaxel, the cell cycle is interrupted in the mitotic phase. The specific binding of paclitaxel molecules (*C*_*SB*_) to the intracellular microtubules determines their stabilization and impedes their reorganization essential for the cellular division. Part of the paclitaxel exchanged from the extracellular (*C*_*F*_) to the intracellular ambient (*C*_*i*_) may remain unbound.

To describe the transport of paclitaxel within the arterial tissue, a transient diffusion-reaction model was defined in accordance with previous studies [18, 23]. In the absence of realistic values of the pressure gradient induced by DCB inflation across the vessel wall, the contribution of advection to drug transport within the wall was neglected. The interaction of paclitaxel with the components of the vessel wall was modelled as a non-linear, saturable, reversible reaction term [24]. In detail, the reversible binding of ligands (i.e. the free paclitaxel molecules) with receptors (i.e. the specific intracellular receptors, corresponding to the microtubules [25]) leading to the formation of a ligand-receptor complex was described. This biological process, being reversible, is governed by the association and dissociation rate constants, which are related to tissue properties. Fig 2 shows a schematic representation of the biological processes and current understanding of the action of paclitaxel. Only the reversible and saturable specific binding was integrated in this model, neglecting the non-specific binding of paclitaxel (namely the binding of the paclitaxel molecule to various extracellular matrix components [15]). This is biologically translated into the exertion of the effect (i.e. inhibition of the cellular proliferation or migration) by the specific intracellular receptors, which leads to the arrest of the cellular mitosis (as schematically represented in Fig 2). Moreover, the extracellular and intracellular free drug concentrations were assumed to be equal at all times, an approximation that is valid when the transport across the cell membrane is sufficiently fast. Therefore, the overall mathematical model of paclitaxel kinetics in the SFA wall was described through the following system of equations:

$$\left\{ \begin{array}{l} \frac{\partial C_{F}}{\partial t}=\nabla ∙(D_{eff}\nabla C_{F})-\frac{\partial C_{SB}}{\partial t}\\ \frac{\partial C_{SB}}{\partial t}=k_{on}[C_{i}(b_{max}-C_{SB})-k_{D}C_{SB}]\\ C_{i}=C_{F} \end{array}\right.$$ (1)

where *C*_*F*_ and *C*_*i*_ represent the extracellular and intracellular concentration of the free paclitaxel (i.e. not bound to receptors), respectively; *D_eff_* is the effective diffusion coefficient (which varies between the healthy and calcified vessel portions); *C_SB_* is the concentration of the specifically-bound drug; *k_on_* and *k_D_* are the association and dissociation rate constants, respectively and; *b_max_* is the maximum concentration of binding sites (i.e. active receptors available to uptake paclitaxel, assumed to vary between the healthy and calcified portions) [26]. Summarizing, the paclitaxel kinetics were described as a function of the free (*C*_*F*_) and the specifically-bound drug concentrations (*C*_*SB*_). Table 1 summarizes the parameters of the mass transport model, with the related values.

**Table 1:** List of the parameters of the drug transport model.

Parameter	Ref.	Value	Description
*D*_*eff*, *H*_	[22]	5.41·10^−5^ mm^2^/s	Effective diffusion coefficient in the healthy wall
*D*_*eff*, *C*_	[21]	1.99·10^−7^ mm^2^/s	Effective diffusion coefficient in the diseased wall
C_0_	*	3.5 μg/mm^2^	Surface concentration of paclitaxel for the IN.PACT Admiral balloon
*b*_*max*,*H*_	[18]	4.2 mmol/L	Maximum density of the binding sites in the healthy wall
*b*_*max*,*C*_	[21]	1.3 mmol/L	Maximum density of the binding sites in the diseased wall
*k*_*D*_	[25]	5·10^−6^ mmol/m^3^	Specific equilibrium dissociation constant
*k*_*on*_	[27]	8·10^11^ mm^3^ / (mol s)	Specific drug binding rate constant
*k*	**	6.5·10^−7^ kg / (m^2^ s)	Rate constant to define an exponential decay of drug concentration on the lumen wall in contact with the balloon

* IN.PACT^TM^ Admiral^TM^ drug coated balloon (Medtronic) instruction for use

** Estimated from experimental considerations [30].

#### 2.2.2 Boundary conditions

With reference to Fig 1B, the lumen wall in contact and not in contact with the DCB were denoted *Γ*_*BALL*_ and *Γ*_*LUMEN*_, respectively, the adventitial wall was denoted *Γ*_*ADV*_, and the lateral opening of the vessel was denoted *Γ*_*OP*_. Following a previous work [28], a zero-flux condition was applied to *Γ*_*OP*_: due to the large distance from the region of interest, this choice of boundary condition had negligible impact on the results. A perfect sink condition was applied to *Γ*_*ADV*_ [18]. As a first approximation, the drug was not allowed to enter the *Γ*_*LUMEN*_, and thus a zero-flux condition was applied. While all the other boundary conditions were kept constant, at the beginning of the DCB inflation the drug concentration on *Γ*_*BALL*_ was set equal to the value of the initial concentration present on the DCB surface, assuming a large excess of paclitaxel (3.5 μg/mm^2^, as declared in the instruction for use of IN.PACT Admiral device). After DCB application, to mimic the removal of the balloon and the wash-out of the blood, the drug concentration on *Γ*_*BALL*_ was set to 0. In an attempt to understand the impact of coating retention on the lumen wall, we also considered the case of an exponential decay of drug concentration on *Γ*_*BALL*_ [29] after removal of the balloon. The exponential decay, defined as a negative, concentration-dependent flux, included a rate constant *k* (Table 1), which was derived from previous experimental considerations [30]. In particular, it was assumed that more than half of the coating load was lost in the first 20 minutes after balloon deflation.

#### 2.2.3 Numerical solver

The mathematical model described in the system of Eq (1) was solved using the commercial software CFX (v.18.2, Ansys Inc., Canonsburg, PA, USA). The solver settings are summarized in Table 2.

**Table 2:** List of the solver settings.

Type	ANSYS CFX–pressure-based
Pressure-velocity coupling method	Coupled
Spatial discretization scheme–gradient	Least squares cell based
Spatial discretization scheme–pressure	Second order
Spatial discretization scheme–momentum	Second order upwind
Transient scheme	Second order Backward Euler
Convergence control (max coefficient loops)	5
Convergence criterion for the global residuals	10^−6^
Time-step size	10 seconds

### 2.3 Investigated scenarios

Based on the guidelines of the IN.PACT Admiral DCB, which suggests a balloon dilatation time of 180 seconds for obtaining an optimum mechanical dilatation and a minimum time for adequate drug transfer of 60 seconds, different clinical scenarios were considered for this study. In detail, the effect on drug uptake and distribution was investigated when:

- DCB inflation time was varied (i.e. 60, 120 and 180 seconds), for a single DCB application;
- sequential application of two DCBs was considered. An inflation time of 60 seconds was simulated for the first DCB, followed by a clinically realistic interlude of 5 minutes before a further inflation of a second fully loaded DCB for a time of 60 seconds. This double DCB application (total inflation time of 60+60 seconds) was compared to the corresponding single DCB application of 120 seconds;
- the coating was assumed to be retained on the lumen wall for a period of time after inflation. The results of a single DCB application with 60-, 120- and 180-second inflation time performed with the presence of the coating (i.e. exponential decay applied on *Γ*_*BALL*_) were compared to the analyses with an efficient blood wash-out (i.e. null concentration applied on *Γ*_*BALL*_).

### 2.4 Analysis of the results

For each investigated scenario, the free (*C*_*F*_) and the specifically-bound (*C*_*SB*_) drug concentrations were compared immediately after DCB inflation (60, 120 or 180 seconds) and then subsequently 10 minutes and 1 hour later. The temporal profiles of *C*_*F*_ and *C*_*SB*_ were analyzed across the wall thickness in the healthy Ω_HEAL_ and the calcific Ω_CALC_ regions of the diseased vessel wall. To simplify the comparison, the drug concentrations were normalized by the maximum concentration value (relative to Ω_HEAL_), for both *C*_*F*_ and *C*_*SB*_ concentrations. The free drug was maximal during the balloon inflation in the proximity of *Γ*_*BALL*_. Thus, *C*_*F*_ was normalized by that value. On the contrary, *C*_*SB*_ was normalized by the final value of the longest DCB exposure (i.e. 180 seconds) observed at 1 hour after the DCB deflation.

## 3. Results

### 3.1 Impact of DCB inflation time

**Fig 3:**
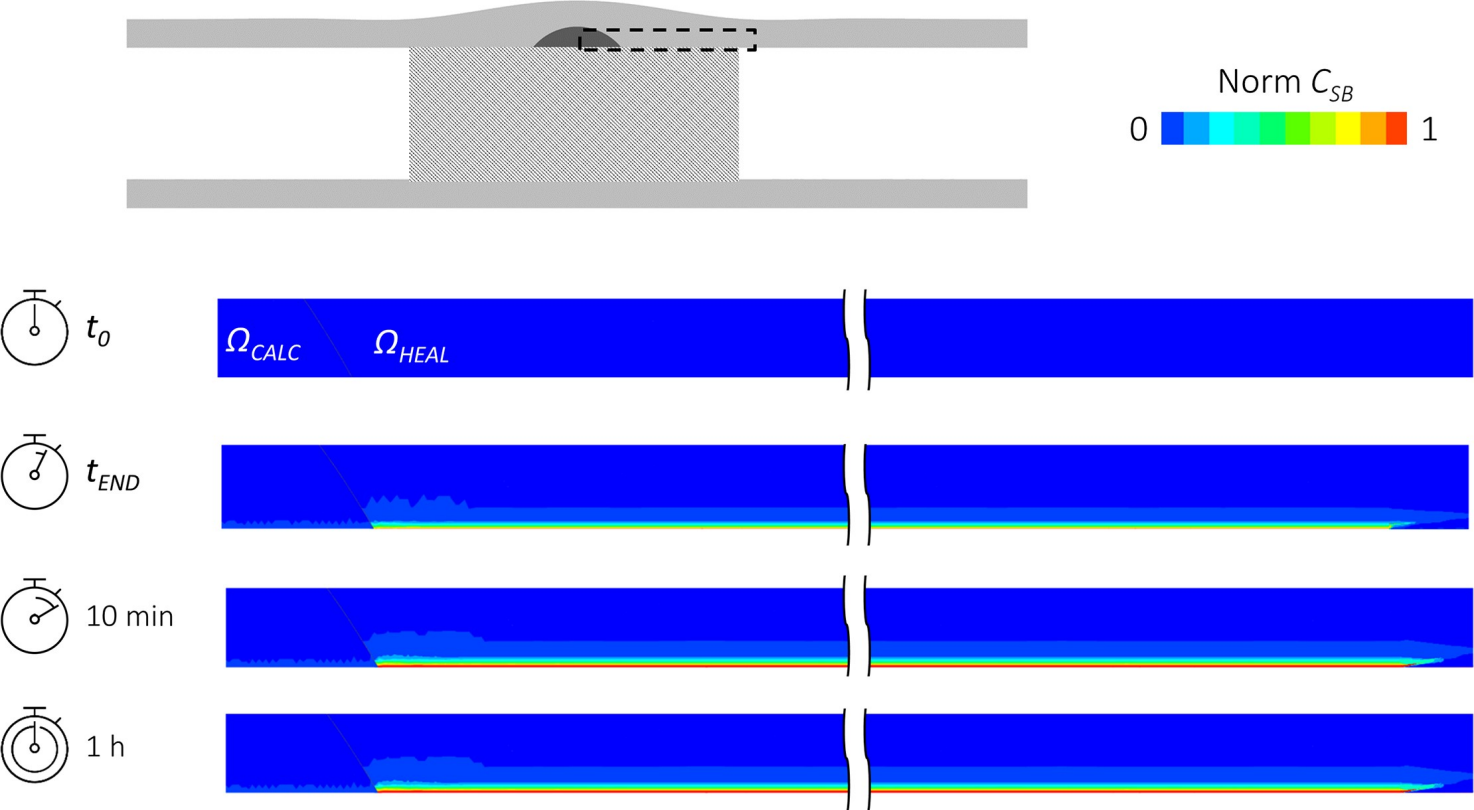
Spatial maps of normalized specifically-bound (*C*_*SB*_) drug concentration on the section along the main direction in the case of DCB inflation of 180 seconds. The temporal evolution is studied at 4 time instants: the beginning (*t*_*0*_) and end (*t*_*END*_) of the DCB inflation, at 10 minutes (10 min) and 1 hour (1h) after DCB deflation. The location of the color maps is indicated at the top of the schematic.

Fig 3 shows contour maps of the normalized specifically-bound (*C*_*SB*_) drug concentrations, following the 180-second exposure-time. The drug concentration was captured in 2D on both the diseased and healthy portions of the lumen wall along the main vessel direction at 4 time instants: the beginning and end of the DCB inflation (*t*_*0*_ and *t*_*END*_), at 10 minutes and 1 hour after DCB deflation. It is qualitatively observable that, due to the lower diffusivity of drug in the calcific region *Ω*_*CALC*_, drug penetration was limited in *Ω*_*CALC*_ as compared to *Ω*_*HEAL*_. Moreover, the drug concentrations at 10 minutes and 1 hour after DCB deflation were almost identical, highlighting the strong retention of the drug in the tissue. The penetration front of *C*_*SB*_ progressively moved towards the adventitia, following the free drug (*C*_*F*_) distribution (not shown).

**Fig 4:**
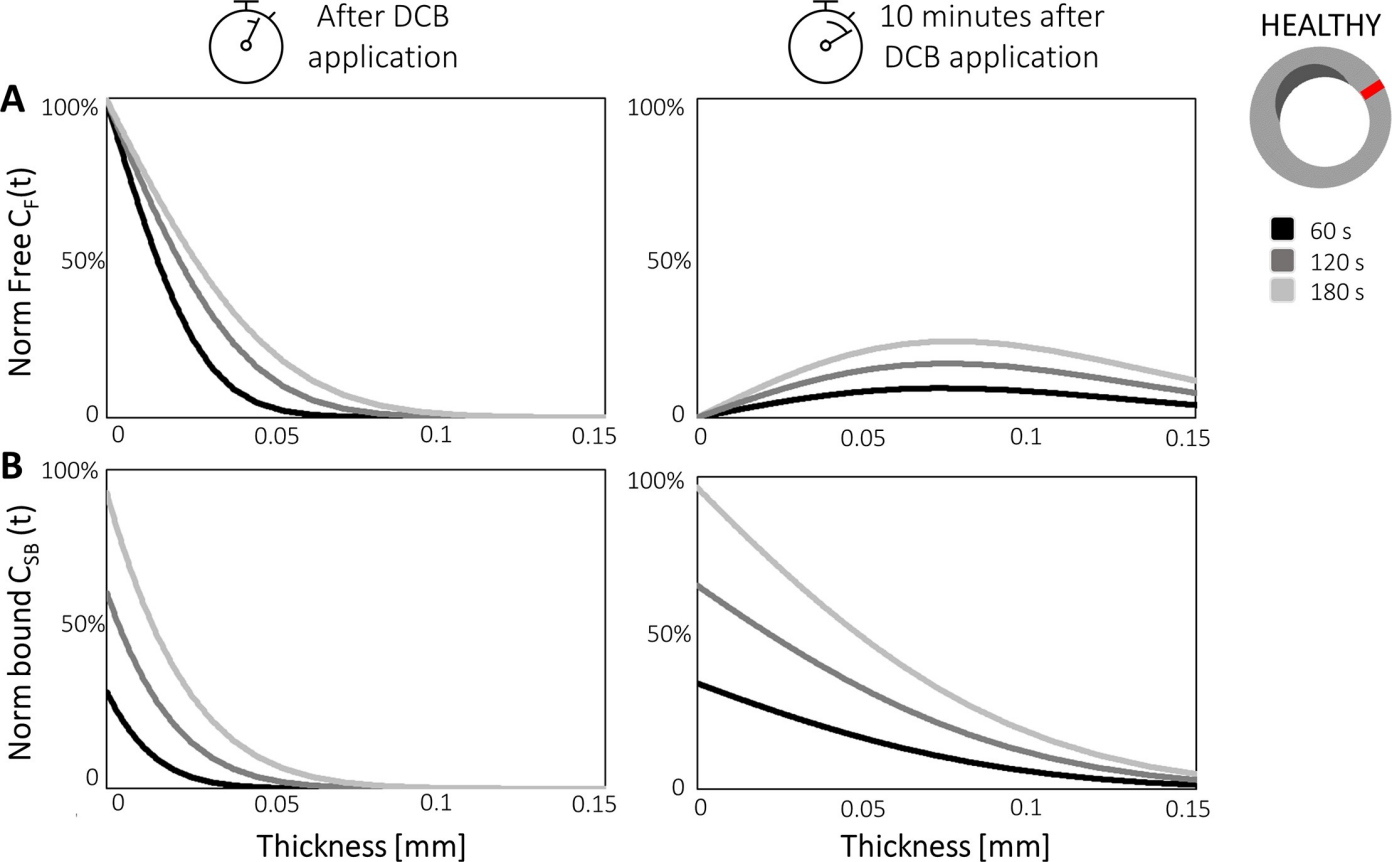
Impact of DCB inflation time on normalized free *C*_*F*_ **(A)** and bound *C*_*SB*_ **(B)** drug concentrations along the vessel wall thickness in healthy regions *Ω*_*HEAL*_. Results are displayed for inflation times of 60, 120 and 180 seconds. The location of the profile analyzed is indicated on the cross-section.

**Fig 5:**
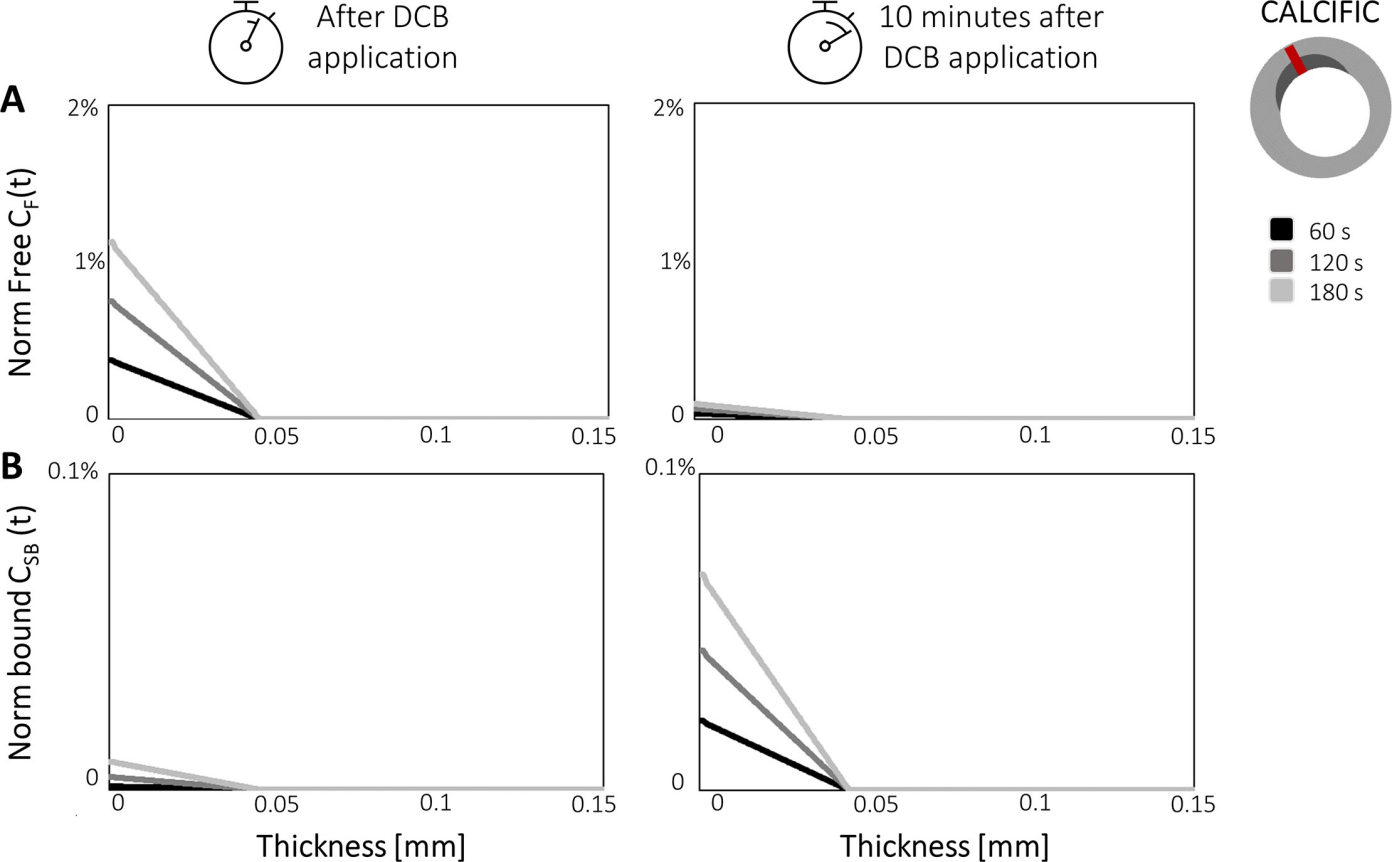
Impact of the DCB inflation time on normalized free *C*_*F*_ **(A)** and bound *C*_*SB*_ **(B)** drug concentrations along the vessel wall thickness in the calcific regions *Ω*_*CALC*_. Results are displayed for inflation times of 60, 120 and 180 seconds. The location of the profile analyzed is indicated on the cross-section.

To gain a more quantitative insight, spatial profiles of *C*_*F*_ and *C*_*SB*_ along a line extending from the lumen to the adventitia are shown for the three different inflations times for both the healthy (Fig 4) and calcific tissues (Fig 5). Results are shown immediately after DCB inflation and 10 minutes after DCB deflation. Considering firstly the comparison at the end of the DCB inflation, the drug penetration front for the longest exposure (180 seconds) reached approximately 100 μm in the healthy portion (Fig 4), while in the calcific region the drug penetrated only 50 μm (Fig 5), due to the lower diffusivity exhibited in *Ω*_*CALC*_. Focusing on *Ω*_*HEAL*,_ differences up to ~30% in *C*_*F*_ were observed between the 60- and 180-second exposures (Fig 4A). Considering *C*_*SB*_, the largest difference between 60-second and longest 180-second DCB inflations was visible in proximity of the lumen wall, where the free concentration was maximal (Fig 4B). Despite the non-linearity of the specific binding kinetics, the maximal *C*_*SB*_ value for an inflation time of 180 seconds was almost three-fold the corresponding value for a 60-second inflation.

The maximum value of *C*_*F*_ observed for the 180-second exposure was approximately 4 orders of magnitude lower in *Ω*_*CALC*_ (Fig 5A) compared with *Ω*_*HEAL*_ (Fig 4A)_._ Analogous findings emerged for the bound drug (Figs 4B and 5B). Moreover, the reduction in the maximal value of bound drug with decreasing inflation time in *Ω*_*CALC*_ was non-linear, possibly explained by the 3 orders of magnitude lower binding site density compared with the healthy tissue.

Analyzing the drug concentration profile at 10 minutes after the DCB application, a significant reduction of the free drug was observed in both *Ω*_*HEAL*_ and *Ω*_*CALC*_ particularly near the lumen wall where blood wash-out conditions were imposed (on *Γ*_*BALL*_) (Figs 4A and 5A). By this time, the drug had penetrated the full wall thickness in *Ω*_*HEAL*_ (Fig 4A), while the penetration distance in *Ω*_*CALC*_ remained limited to 50 μm, owing to the presence of the calcification (Fig 5A). At the lumen wall, due to the fast binding of paclitaxel, *C*_*BS*_ was maximum for the case with 180-second exposure (Figs 4B and 5B). However, in the wall tissue far from the lumen wall, the *C*_*BS*_ penetration distance more than trebled in 10 minutes as a consequence of increased *C*_*F*_ penetration (Fig 4B). The diffusion-limited behavior of paclitaxel kinetics can be better observed in the calcific portion where the distribution of *C*_*BS*_ (whose concentration increase 5-fold in 10 minutes, Fig 5B) declined rapidly, as with the free drug concentration.

At 1 hour after DCB application, the free drug concentrations *C*_*F*_ were negligible in the vessel wall (results not shown). Furthermore, negligible differences (lower than 0.3%) in terms of *C*_*SB*_ emerged between the 10-minute and 1-hour distributions (Fig 3). This was a consequence of the rapid withdrawal of the free drug by the blood wash-out, which limited the amount of free drug that could subsequently be uptaken into the intracellular region and made available for binding to the microtubule receptors.

Given the negligible drug concentrations in the *Ω*_*CALC*,_ in the following sections the results are shown for the *Ω*_*HEAL*_ region only. Instead, the results for the *Ω*_*CALC*_ are reported in the S1 File (Section “Supplementary results”) for the sake of completeness.

### 3.2 Impact of single vs. double DCB application

**Fig 6:**
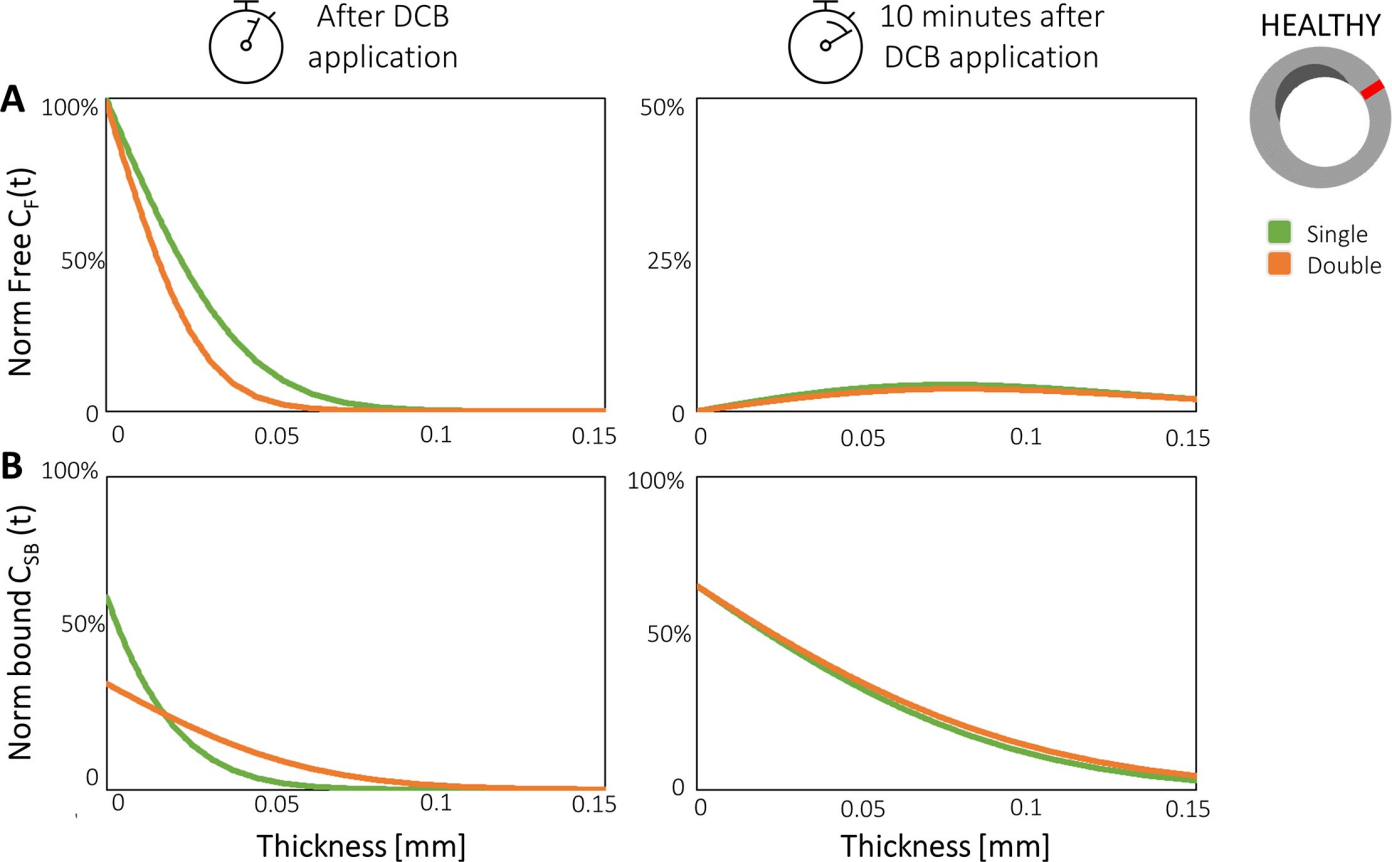
Results of the sequential application of multiple DCBs. Profiles along the wall thickness immediately after and 10 minutes after DCB application. Plots display **(A)** free (*C*_*F*_) and **(B)** specifically-bound (*C*_*SB*_) drug concentrations for the healthy region for the single (green line) and double (orange line) DCB applications. *C*_*F*_ was normalized by the initial values (initial drug loading, 3.5 μg/mm^2^); *C*_*SB*_ was normalized by the maximum measured values, namely at 1 hour from the DCB removal.

Fig 6 depicts the spatial profiles of the normalized free *C*_*F*_ and bound *C*_*SB*_ drug concentrations in the healthy *Ω*_*HEAL*_ regions of the treated portion of the lumen wall, comparing the single with the sequential double DCB application (with the same total exposure time of 120 seconds). As expected, *C*_*F*_ and *C*_*SB*_ displayed similar distributions in *Ω*_*HEAL*_ between the single and double DCB applications. Comparing the results at the end of the procedure (Fig 6, left column), the values of *C*_*F*_ were lower in the double DCB application compared with the single application, with a maximum percentage difference of ~15% at a depth of 0.035 mm from the lumen wall (Fig 6A). This follows from the shorter (60 seconds) application of the second loading, which took place after the interlude of 5 minutes, interval long enough to consent a complete removal of the free drug in the tissue. Since the single application presented a continuous exposure for a longer time (120 seconds), at the end of the procedure *C*_*F*_ resulted higher in the tissue (Fig 5A). The values of *C*_*SB*_ were lower (~38%) in the proximity of the lumen wall in case of the double DCB application, due to the loss of free drug during the “interlude” between the two inflations (Fig 6B). This effect was caused by the rapid blood wash-out that limited the quantity of drug available for the specific binding. However, moving towards the adventitia (e.g. at a depth of 0.040 mm from the lumen wall), *C*_*SB*_ was ~12% higher in the double DCB application case, suggesting that the interlude phase promoted increased binding in the internal tissue (Fig 6B).

Concerning the results at 10 minutes after DCB application (Fig 6, right column), the differences in terms of free and bound drug were negligible between the two scenarios, because of a larger availability of *C*_*F*_ initially present after the longer single DCB application (Fig 6A). For this reason, *C*_*SB*_ was also comparable between the two scenarios, even if slightly larger in case of the double DCB application (maximum difference < 5%, Fig 6B).

### 3.3 Impact of DCB coating retention

**Fig 7:**
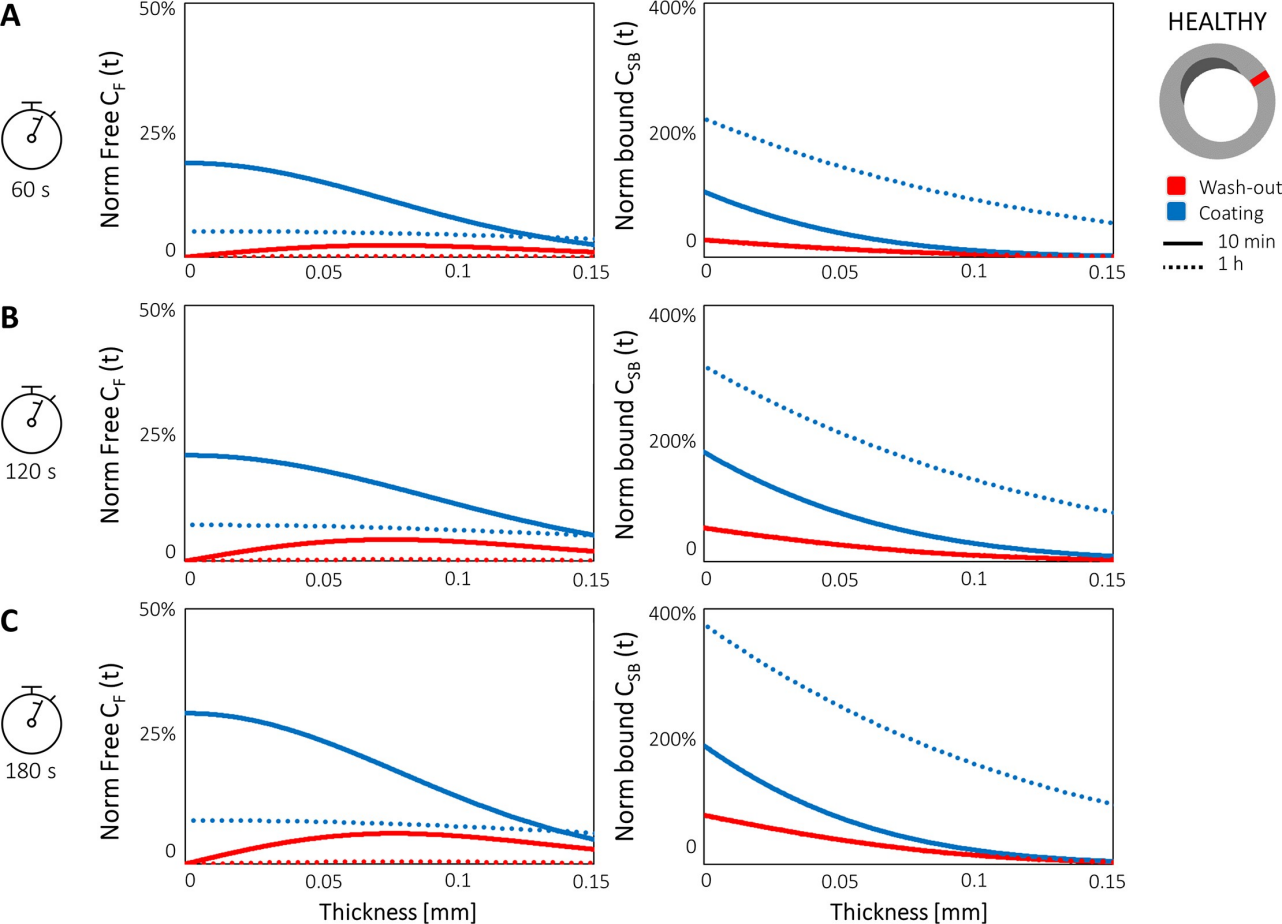
Simulations of DCB inflation for **(A)** 60, **(B)** 120, and **(C)** 180 seconds in case of efficient blood wash-out (red) and coating retention (blue). The spatial profiles of the free *C*_*F*_ and bound *C*_*SB*_ drug concentrations are compared at 10 minutes (continuous line) and at 1 hour (dotted line) after DCB application. Both *C*_*F*_ and bound *C*_*SB*_ were normalized by their relative maximum values. *C*_*F*_ was normalized by the initial values (initial drug loading); *C*_*SB*_ was normalized by the maximum measured values, namely at 1 hour after the DCB removal. When not visible, the red dotted line is overlapped to red solid line.

Fig 7 illustrates the spatial profiles of *C*_*F*_ and *C*_*SB*_ drug concentrations following inflations of 60 (Fig 7A), 120 (Fig 7B) and 180 (Fig 7C) seconds, examined at 10 minutes and 1 hour after DCB application. From the comparisons performed between the coating retention and blood wash-out cases, it emerged that the drug kinetics were not affected during the DCB inflation and the spatial profiles of *C*_*F*_ and *C*_*SB*_ at the end of DCB application were identical (not shown). The impact of coating retention is evident beginning from the comparison at 10 minutes. Indeed, in the case of coating retention, *C*_*F*_ lost ~80% of the initial load in the proximity of the lumen wall (for the 60 second exposure) but was still 3.5-fold larger than in case of efficient blood wash-out (Fig 7A). As established in Section 3.1, the longer the initial DCB inflation, the larger the amount of free drug remaining in the tissue, though the 3.5-fold difference was maintained between the coating retention and wash-out cases.

The retention of the coating also induced notable differences in the profiles of *C*_*SB*_ along the wall thickness. In fact, with the coating retention the maximum concentration of *C*_*SB*_ following the 60-second inflation evaluated at 10 minutes reached the values observed in case of efficient blood wash-out evaluated at 1 hour after the 180-second DCB application (see Fig 7A and Section 3.1). Coherently, the maximal value of *C*_*SB*_ at 1 hour was 10-fold higher assuming coating retention compared with the assumption of blood wash-out. Similar trends were observed in the cases of 120- and 180-second exposure time (Fig 7B and 7C, respectively). As previously observed (Section 3.1), the longer the DCB inflation, the higher the observed bound drug concentrations in the tissue. However, the maximum differences of ~6% between the 60- and 180-second exposure cases found on the lumen wall were negligible as compared to the variations introduced by the coating (Fig 7A and 7C). Indeed, the retention of the coating, even if rapidly decaying in time, increased the retention of paclitaxel, and, consequently, the quantity of *C*_*F*_ available for specific binding. For this reason, despite 85% of the drug coating being lost by 1 hour, *C*_*SB*_ still increased up to 4-fold (in the case of 180-second exposure time, Fig 7C) compared to the efficient blood wash-out scenario.

## 4. Discussion

In this computational study, a 3D mass transport model was proposed to describe drug delivery from a paclitaxel DCB for the treatment of diseased SFAs. Several features were investigated including the impact of different procedural aspects (inflation time, multiple DCB application), pathologies (healthy vs. calcified wall) and modelling assumptions (drug wash-out by the blood stream vs. coating retention). The key findings of the work can be summarized as follows: (i) the DCB inflation time impacts not only the free drug concentrations in the arterial wall, but most importantly the amount of specifically-bound drug; (ii) specific binding of paclitaxel is greatly reduced in calcific plaques, as compared to healthy portions of the wall, primarily as a result of significantly reduced drug diffusion; (iii) a sequential application of two fully-loaded DCBs leads to modest differences in drug concentration in the inner part of the wall immediately after inflation, but the differences rapidly become negligible (within 10 minutes); and (iv) retention of the DCB coating may lead to notable variation of the drug free and specifically-bound drug concentrations in the vessel wall.

In contrast to the vast literature considering computational modeling of the drug eluting stents (e.g. [28, 31–33]), where drug delivery is typically sustained over a period of weeks, the number of published studies considering computational modeling of DCBs (and specifically for diseased SFAs) is extremely low [15–18]. Given the limited time of contact between the DCB external surface and the arterial wall, an adequate understanding of drug transport and retention in a realistic arterial model and of the impact of procedural aspects becomes of extreme importance for the success of DCB treatment for diseased SFAs [34].

The main methodological novelty of this work is the extension of the mass transport model to the 3D setting, allowing the drug kinetics to be assessed in more realistic asymmetrical geometrical configurations. The results are particularly interesting for the analysis of the treatment of vascular segments characterized by lesions of unique composition and extent. Furthermore, compared to other anatomical locations, DCB inflation in SFAs may be prolonged for several minutes [35]. In accordance with the recent findings for 2D cross-sectional diseased coronary arteries [15], the longer the DCB application, the deeper the penetration front within the tissue. Moreover, the introduction of the behavior of the paclitaxel within the intracellular environment through specific binding provides interesting insights coherent with previous experimental findings [36], in which paclitaxel retention remained particularly high in the first hour after DCB implantation.

From the findings on the impact of different tissue composition on drug transport, and in accordance with previous literature on DCB computational modelling [15, 16, 18], marked differences in terms of free and bound concentrations were found between the regions *Ω*_*HEAL*_ and *Ω*_*CALC*_. These results reflected the difference in terms of effective drug diffusivity, which was 4 orders of magnitude lower in the diseased region than in the healthy one, and the lower maximum binding sites density [21]. In particular, even after a 180-second DCB inflation, which is suggested on the device instructions for use, the free drug concentrations in the calcific region were 1000-fold lower than in the healthy tissue. Consequently, the diffusion-limited nature of paclitaxel transport [26] dictates that *C*_*SB*_ in Ω_CALC_ was also significantly reduced (approximately 4 order of magnitudes lower as compared to healthy regions). These findings suggest that the mode of action of a DCB, besides the simplicity of the implantation technique as compared to other treatments, might not be trivial. Specifically, in terms of the drug concentrations that may be achieved in the vessel wall, our results indicate that this device may well be highly-effective in healthy vessel tissue, yet possibly ineffective in calcified arteries which are the ones that usually require intervention.

In the current clinical practice, a single DCB application is usually performed. However, to avoid recurrent restenosis, multiple DCB applications may also be performed [10]. From this analysis (in this specific case, a double DCB application), it has been demonstrated that the paclitaxel kinetics are largely unaffected by the interventional procedure. In particular, similar but not identical dynamics in terms of free and specifically-bound paclitaxel were found when comparing the single with the double DCB application with the same total exposure time of 120 seconds.

Lastly, the drug kinetics in presence of an efficient blood wash-out (null concentration applied on *Γ*_*BALL*_) was compared to the case of the DCB coating adhered to the lumen wall and subjected to an exponential decay (exponential decay applied on *Γ*_*BALL*_). Notably, this analysis illustrated that, considering the same inflation time, the retention of the coating on the vessel wall widely affected the specifically-bound drug, trebling or even quadrupling its concentration within the arterial tissue as compared to the blood wash out condition. This result is in agreement with recent experimental studies and suggests that more sophisticated modelling of coating retention should be considered in future [29, 30].

The findings of this work have a general validity, as an idealized model was created comparing different scenarios by changing one feature at a time while maintaining all the other characteristics. The model developed here is characterized by a wide versatility that, for example, could also allow replacement of the idealized geometry with a patient-specific geometrical model (e.g. reconstructed from clinical images as previously performed on 2D sections [16]), with the final aim of exploring personalized DCB applications. Furthermore, this 3D setting also enables the integration of structural simulations of DCB application, which may provide realistic values of the pressure gradient across the vessel wall to facilitate the realistic incorporation of interstitial flow. Perhaps more importantly, the state of deformation of the wall tissue could also be introduced, information that could be, in principle, compared with observed *in-vivo* application through imaging techniques. To date, in the available computational DCB literature, only the pressure gradient has been introduced (e.g. [15]), without the introduction of the state of deformation induced in the arterial wall during the DCB application.

Finally, we wish to reiterate that although this work has produced interesting results, these have to be considered within the context with the limitations of the model. Firstly, the idealized model adopted in this work presents a single wall layer with homogeneous properties and presenting with only two materials (i.e. healthy tissue and a calcific plaque). As recently studied in a 2D mass transfer model of a stented coronary artery by Escuer et al. [28], different material properties could be added to describe the intimal, medial and adventitial layers separately, as well as the membranes at their interface. However, each additional component must be characterized by diffusive and binding properties, whose values are not readily available in the literature in the case of SFAs, especially for patient-specific cases. With the ultimate goal of investigating patient-specific cases, the introduction of an excessive level of detail would lead to an overcomplication of the computational model, impeding a reliable though simpler analysis of the major features influencing the drug kinetics. Moreover, although diffusion is commonly assumed to be the dominant mechanism during drug release and uptake [18, 23], the porous nature of the vessel wall and the pressure gradient across the wall imposed during angioplasty may contribute to a non-negligible advective term [15]. Hence, to further improve this mathematical model, which accounts only for the diffusion and reaction terms, the advection term could be incorporated. However, in a real clinical case scenario, this term should reflect not only the pressure gradient induced during the angioplasty procedure, but also the changes in the arterial morphology and thus the geometrical model. Lastly, the wash-out of the DCB coating was described here through a simplistic exponential decay. However, this aspect of the DCB mode of action should be more deeply analyzed. In particular, given the very recent findings regarding DCB excipient technologies [9, 29], the different releasing mechanisms from various coating microstructures (e.g. needle-shaped or spherical particles) of the DCB could be implemented in a more sophisticated model.

## 5. Conclusions

This work presented a 3D computational mass transport model to investigate the mode of action of paclitaxel DCB therapy in diseased SFA. Despite some simplifications, the present work is the first study, to the best of authors’ knowledge, to investigate within a 3D setting, the impact of sequential DCB application as well as of coating retention on diseased SFAs. The findings suggested that the kinetics and thus the biological effects exerted by paclitaxel may be affected not only by the geometrical and compositional features of the treated vessel, but also by the balloon design characteristics and interventional procedural aspects that should be carefully considered. The proposed model is versatile and could ultimately be adapted to patient-specific geometries accounting for different vessel wall layers and plaque components.

## Supporting information

S1 File Analytical validation and sensitivity analysis, and supplementary results. (PDF)
