# Supplementary material for: 3D modelling of drug-coated balloons for the treatment of calcified superficial femoral arteries
Source: PLoS One. 2021 Oct 11;16(10):e0256783. doi: 10.1371/journal.pone.0256783 (PMC8504744; doi:10.1371/journal.pone.0256783)
Supplement: S1 File — (PDF) [file pone.0256783.s001.pdf]

3D modelling of drug-coated balloons for the treatment of calcified superficial femoral arteries

Running title: 3D modelling of DCBs for diseased SFAs

Monika Colombo[1], Anna Corti[1], Scott Berceli[2,3], Francesco Migliavacca[1], Sean McGinty[4], Claudio Chiastra[1,5*]

1. Laboratory of Biological Structure Mechanics (LaBS), Department of Chemistry, Materials and Chemical Engineering "Giulio Natta", Politecnico di Milano, Milan, Italy

2. Malcom Randall VAMC, Gainesville, Florida, USA

3. Department of Surgery, University of Florida, Gainesville, Florida, USA

4. Department of Biomedical Engineering, University of Glasgow, Glasgow, UK

5. PoliTo[BIO]Med Lab, Department of Mechanical and Aerospace Engineering, Politecnico di Torino, Turin, Italy

* Corresponding author

E-mail: claudio.chiastra@polito.it (CC)

SUPPLEMENTARY MATERIALS

Analytical validation and sensitivity analysis

Before performing a sensitivity analysis on the impact of the mesh size and temporal discretization on the numerical results, to gain confidence in the numerical implementation, a comparison was made between the numerical results and the analytical solution of a simplified drug transport model involving only linear diffusion within a healthy arterial wall. Exploiting radial symmetry, the relevant one-dimensional model in a cylindrical co-ordinate system is given by:

$$\frac{\partial c(r,t)}{\partial t} = D\left(\frac{\partial^2 c(r,t)}{\partial r^2} + \frac{1}{r}\frac{\partial c(r,t)}{\partial r}\right), \qquad a < r < b, \qquad t > 0,$$

$$c = c_0, \qquad r = a, \qquad t > 0,$$

$$c = 0, \qquad r = b, \qquad t > 0,$$

$$c = 0,\ a < r < b,\ t = 0, \qquad \text{(S1)}$$

where c represents the free drug concentration, D is the isotropic diffusion coefficient of the drug in the tissue, a and b are the inner and outer radii of the arterial wall, respectively, and c_0 is the constant drug source concentration imposed on the inner wall. Under these conditions, the analytical solution of the drug concentration as a function of time and radial distance from the source is readily shown to be:

$$c(r,t) = c_0\,\frac{\ln(\frac{r}{b})}{\ln(\frac{a}{b})} - 2ac_0\,\sum_{n=1}^{\infty}\frac{\sqrt{\lambda_n}W_n(\sqrt{\lambda_n})U_n(\sqrt{\lambda_n},r)e^{-\lambda_n Dt}}{b^2\lambda_n V^2{}_n(\sqrt{\lambda_n})-4/\pi^2} \qquad \text{(S2)}$$

where the first term represents the steady component and the second term accounts for the transient part of the solution. The transient component depends on an infinite summation and three functions W_n , U_n and V_n, which involve zero- and first-order Bessel functions of the first and second kind. In detail,

$$U_n(\sqrt{\lambda_n},r) = J_0(\sqrt{\lambda_n}a)\,Y_0(\sqrt{\lambda_n}r) - Y_0(\sqrt{\lambda_n}a)\,J_0(\sqrt{\lambda_n}r), \qquad \text{(S3)}$$

$$V_n(\sqrt{\lambda_n}) = Y_0(\sqrt{\lambda_n}a)\,J_1(\sqrt{\lambda_n}b) - J_0(\sqrt{\lambda_n}a)\,Y_1(\sqrt{\lambda_n}b), \qquad \text{(S4)}$$

$$W_n(\sqrt{\lambda_n}) = Y_0(\sqrt{\lambda_n}a)\, J_1(\sqrt{\lambda_n}a) - J_0(\sqrt{\lambda_n}a)\, Y_1(\sqrt{\lambda_n}a), \quad \text{(S5)}$$

where λ_n are found by solving:

$$J_0(\sqrt{\lambda_n}a)\, Y_0(\sqrt{\lambda_n}b) - Y_0(\sqrt{\lambda_n}a)\, J_0(\sqrt{\lambda_n}b) = 0 \quad \text{(S6)}$$

To well-approximate the infinite summation, a small sensitivity analysis on the number of roots λ_n required to achieve sufficient accuracy was carried out using Matlab (v. 2020b, MathWorks, Natick, MA, USA). The number of roots required depends on a number of factors including the values simulated for the wall thickness, the diffusion coefficient and the duration of the phenomenon, which needed to reflect the time frame of the DCB application. The number of roots utilized in the solution computation was $1.2 \cdot 10^6$. The analytical solution was compared with the corresponding solution obtained from simulating the same linear diffusion equation, subject to the same initial and boundary conditions, within a 3D hollow cylinder with internal diameter of 7.3 mm and homogeneous thickness of 1.6 mm (Fig. S1), whose reliability was verified through the analysis of:

i) the grid independence: six different meshes (M_1-M_6), ranging between [8.3÷17.5] million elements, were generated by increasing sequentially the number of elements in the region of interest (Table S1).

ii) the temporal discretization of the transient simulation: three different time-step sizes, namely 1 (TS 1), 10 (TS 2) and 30 (TS 3) seconds, were compared to the analytical solutions.

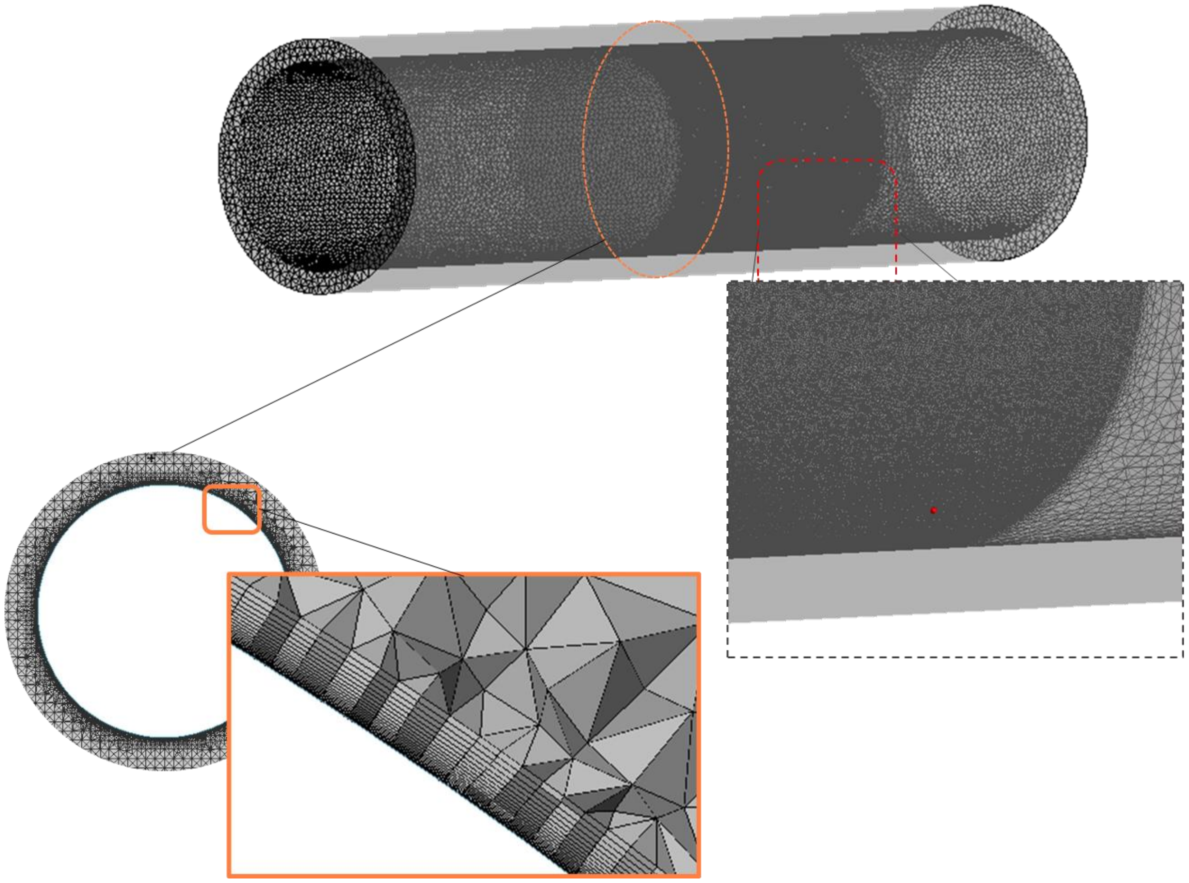

Fig. S1 – Three-dimensional (3D) geometrical model and computational grid of a hollow cylinder, presenting the same lumen diameter and thickness of the healthy portion of the asymmetric model shown in Fig. 1. The central portion of the model presents a denser mesh, where the drug-coated balloon is applied as a boundary condition on the lumen wall, presents a denser mesh. On the bottom left, a detail of the prismatic layers is given.

Table S1. Computational grids adopted for the independence study.

**Name**	**Total elements ($\cdot 10^6$)**	**Boundary layers**	
		**Number**	**Total height**
M_1	8.3	10	0.05
M_2	10.7	15	0.05
M_3	13.1	20	0.05
M_4	13.5	20	0.05
M_5	14.0	20	0.03
M_6	17.5	30	0.05

In both the analyses, the percentage error of the computational results in terms of normalized free drug concentration C(t) was computed with respect to the analytical solutions for a total time of 60 and 120 seconds (Fig. S2). The difference between the computational results and the analytical solution for a total time of 60 seconds (Fig. S2-A) was generally larger than in case of a total time of 120 seconds (Fig. S2-B). However, the percentage error was almost negligible for larger meshes. In fact, as shown in Fig. S3, the results at 60 seconds, were very close in terms of absolute and percentage error when considering a number of 13.1 million elements (9.1%, mesh M_3). The error was almost negligible considering larger meshes (~3%), while it was about 37.9% in case of the coarsest mesh (M_1). For this reason, the parameters adopted for the mesh M_3 were chosen. Similar considerations were made for the temporal discretization analysis (Fig. S4), performed with the previously chosen mesh M_3. In this case, the percentage error induced by a time-step size of 1 s was about 3.4%, against the 9.1% for the time-step of 10 seconds and 17.2% for the time-step size of 30 seconds. However, the absolute error was found to be 0.24, 0.25 and 0.65 for the 1, 10-second and 30-second time-step sizes, respectively. Thus, to reduce the computational effort without losing in accuracy, the time-step size of 10 seconds was chosen.

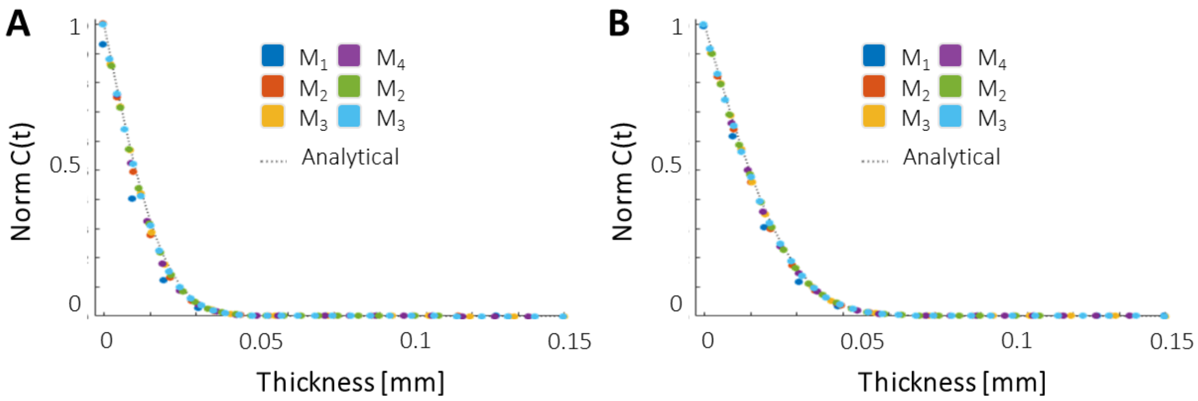

Fig. S2 – Results of the grid independence study. Comparison of the percentage error of the computational results for **A)** 60 and **B)** 120 seconds with respect to the analytical solution for the

grid independence study, represented with the dotted line. M_1 is the coarse mesh, while M_6 is the finer mesh.

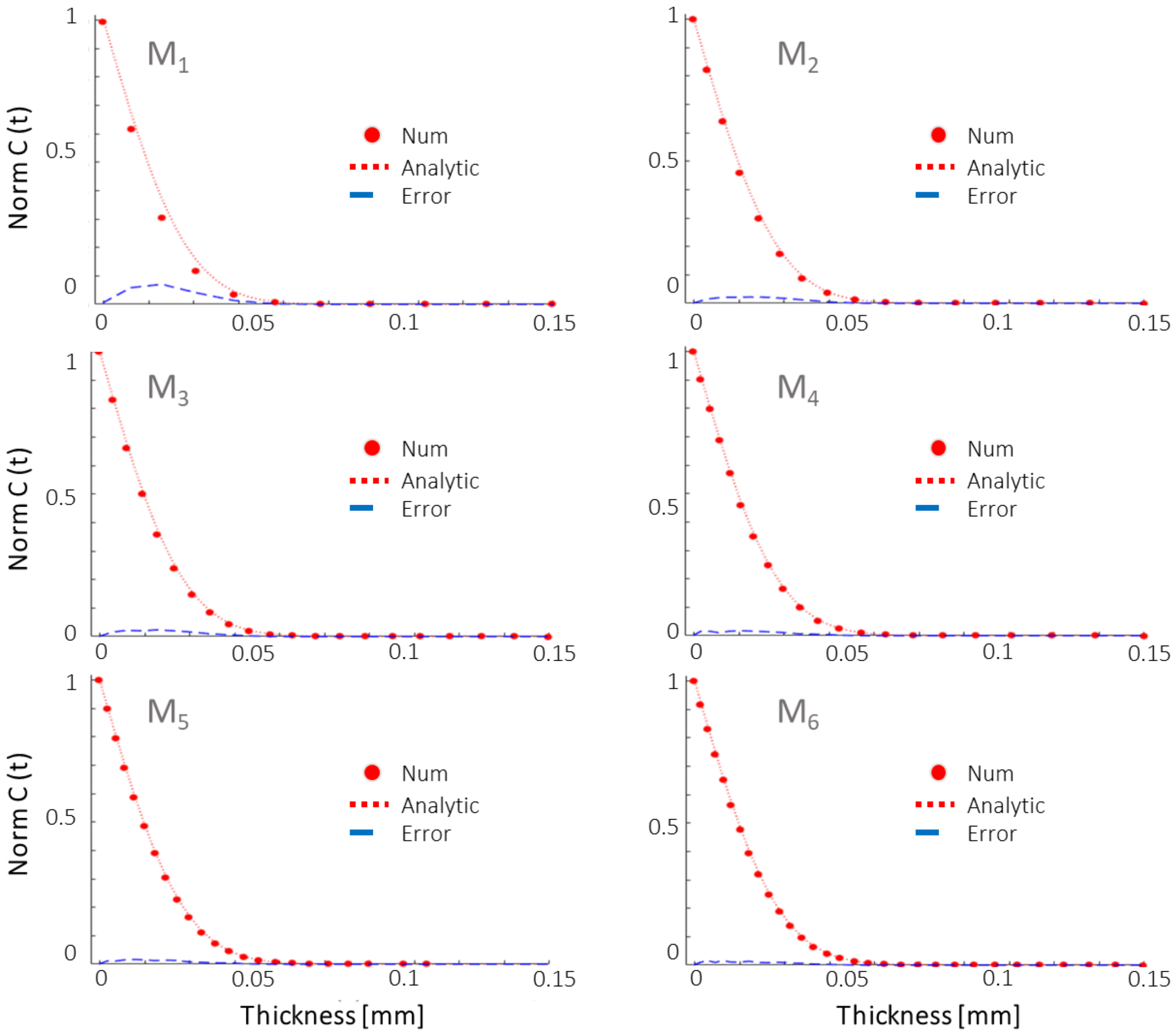

Fig. S3 – Results of the grid independence study. Comparison of the analytical solution (red dashed line) and the numerical results at 60 seconds (red dots). The absolute error computed as the absolute difference between the analytical and the numerical solution is represented with the blue dashed line.

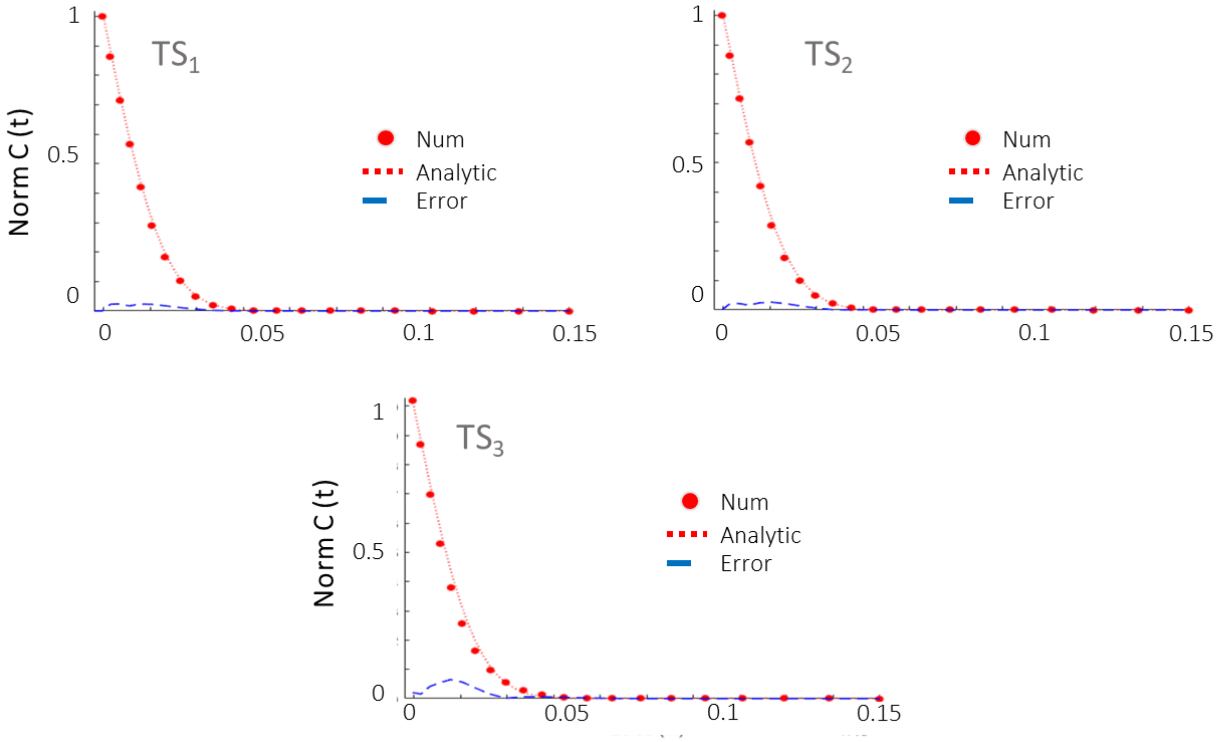

Fig. S4 – Results of the temporal discretization analysis for the 60-second simulation, where TS 1 represents the smaller time-step size (1 second), TS 2 the intermediate time-step size (10 second) and TS 3 the larger time-step size (30 second). The analytical solution is shown as the red dashed line, the numerical results are shown as red dots. The absolute error computed as the absolute difference between the analytical and the numerical solution is represented with the blue dashed line.

Supplementary results: Impact of single vs. double DCB application

Figure S5 illustrates the free C_F and bound C_{SB} drug concentrations observed in the diseased portion Ω_{CALC} for both the single and double DCB application. Negligible differences of C_F (Fig. S5-A) were found at the end of DCB application, mainly due to the low diffusivity in Ω_{CALC}. Analogous findings are observed for the C_{SB} distributions, where slightly larger values (~0.05%) were found at 10 minutes in case of double DCB application (Fig. S5-B).

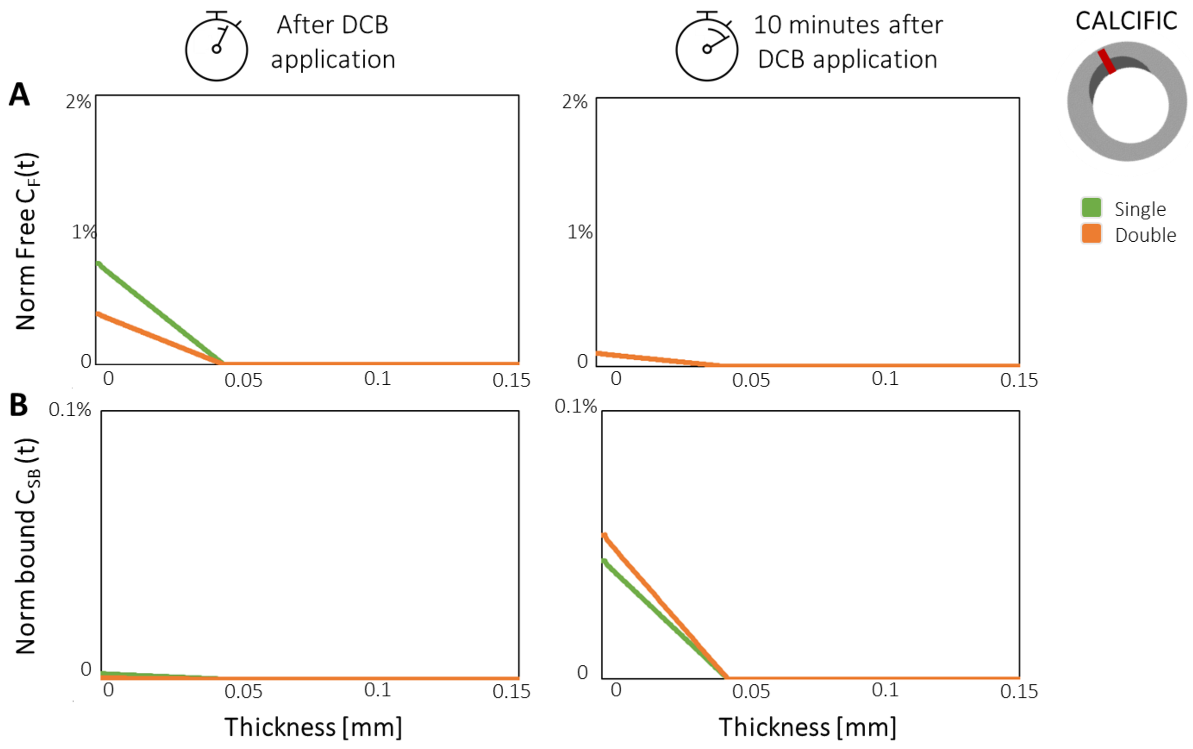

Fig. S5 – Results of the sequential application of multiple DCBs. Profiles along the wall thickness immediately after and 10 minutes after DCB application. Plots display **(A)** free (C_F) and **(B)** specifically-bound (C_{SB}) drug concentrations for the calcific region for the single (green line) and double (orange line) DCB applications. C_F was normalized by the initial values (initial drug loading, 3.5 μg/mm^2); C_{SB} was normalized by the maximum measured values, namely at 1-hour from the DCB removal in the healthy region.

Supplementary results: Impact of DCB coating retention

The DCB coating retention impacted both the free C_F and bound C_{SB} drug concentrations in the diseased portion Ω_{CALC}, as compared to the case of efficient blood-wash out (see Fig. S6), even though less notably as compared to the healthy region. In detail, DCB coating retention determined a respective increase of ~0.1% and ~0.18 % in C_F and C_{SB} at 10 minutes after the 60-second DCB application, as compared to the efficient blood wash-out condition (Fig. S6-A). As expected, this difference, even though negligible as compared to that obtained in Ω_{HEAL}, increased up to ~0.35% and ~0.4% for C_F and C_{SB} at 1 hour after the 180-second DCB application (Fig. S6-C). Coherently with the results for the healthy portion, these findings indicate that DCB coating retention induced a more prolonged availability of free drug in the tissue, thus enhancing the distribution of the specifically-bound drug.

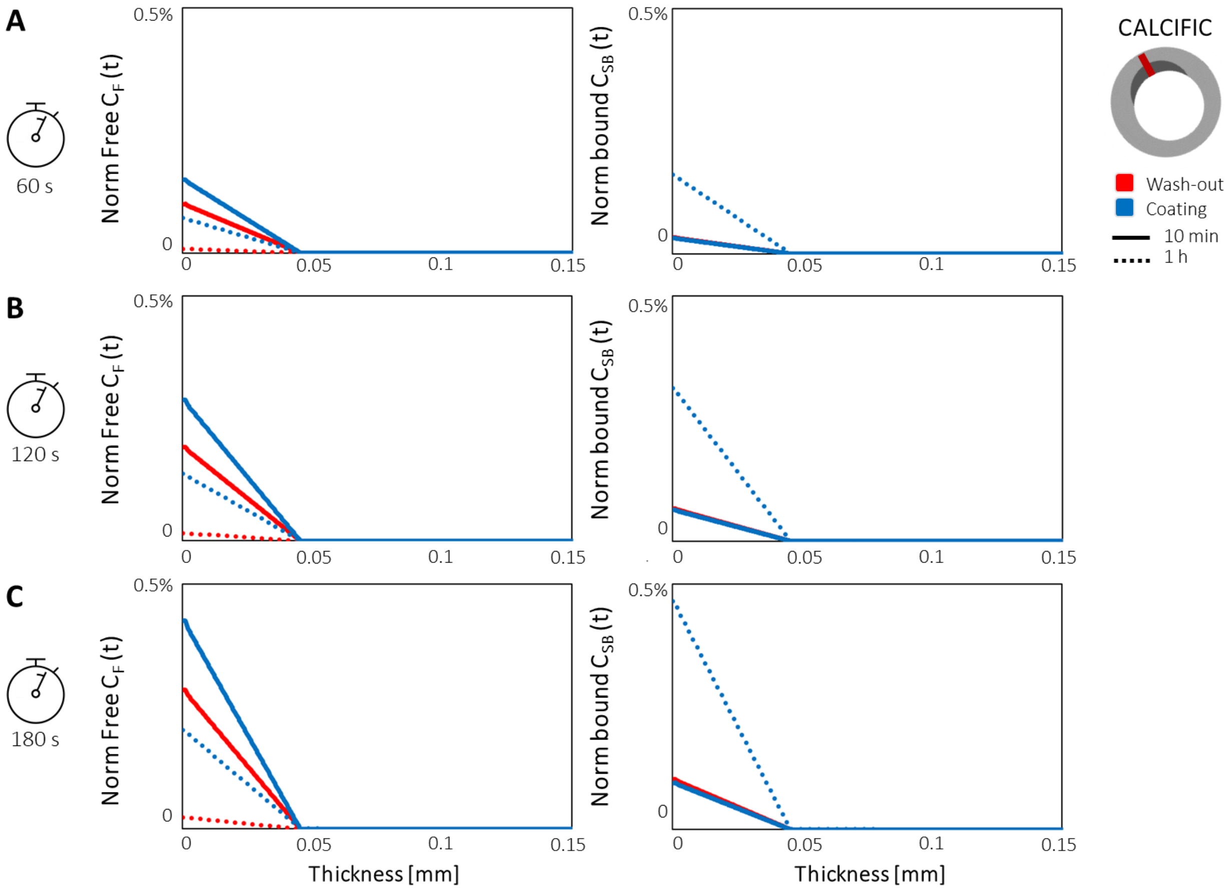

Fig. S6 – Simulations of DCB inflation for **(A)** 60, **(B)** 120, and **(C)** 180 seconds in case of efficient blood wash-out (red) and coating retention (blue) in the diseased region. The spatial profiles of the free C_F and bound C_{SB} drug concentrations are compared at 10 minutes (continuous line) and at 1 hour (dotted line) after DCB application. Both C_F and bound C_{SB} were normalized by their relative maximum values. C_F was normalized by the initial values (initial drug loading); C_{SB} was normalized by the maximum measured values, namely at 1-hour after the DCB removal in the healthy region. When not visible, the red solid and dotted lines are overlapped to blue solid line.
